# Trend of HIV/AIDS for the last 26 years and predicting achievement of the 90–90-90 HIV prevention targets by 2020 in Ethiopia: a time series analysis

**DOI:** 10.1186/s12879-018-3214-6

**Published:** 2018-07-11

**Authors:** Tadele Girum, Abebaw Wasie, Abdulsemed Worku

**Affiliations:** 1 Department of Public Health, College of Medicine and Health Sciences, Wolkite University, Wolkite, Ethiopia; 2 Department of Medicine, College of Medicine and Health Sciences, Wolkite University, Wolkite, Ethiopia

**Keywords:** HIV/AIDS, Antiretroviral therapy, Viral suppression, The 90–90-90 target

## Abstract

**Background:**

HIV infection continues to be epidemic of public health importance with a prevalence of 1.1% and incidence of 0.33/1000 population having low-intensity mixed epidemic. Ethiopia has adopted the 90–90-90 by 2020 target but its progress was not yet assessed. Therefore, this study aimed to assess the trend of HIV infection for the last 26 years and to predict the achievements of the 90–90-90 target.

**Methods:**

We used aggregates of HIV/AIDS indicator data from 1990 to 2016 of UNAIDS data bases. The data were analyzed with excel and STATA. The trend line that best fits the regression was drawn, annual change was estimated and future values of HIV detection rate, coverage of antiretroviral therapy and viral suppression indicators were predicted and compared with the 90–90-90 targets.

**Result:**

Since 1995, new infection has declined by 81% and since 2002; number of HIV cases has declined by 35.5%. However, after remarkable decline for decades, since 2008 HIV incidence rate began to rise by 10% and number of new infection diagnosed each year increased by 36% among all ages and doubled among adults. ART coverage has increased by 90% among all age and tripled among pregnant women within 6 years. Nationally, 67% of people living with HIV know their status, 88% of them are on treatment and 86% of people on treatment have viral suppression. As a result, AIDS-related death declined by 77 and 79% among all age and children respectively. By 2020, 79% of people living with HIV will know their HIV status, of which 96–99% of HIV infected people will be on ART and more than 86% will have viral suppression.

**Conclusion:**

After remarkable decline, HIV infection started to increase in the last few years among adults. With the current trend, Ethiopia will achieve the second and third 90% HIV targets, while the first target is not achievable and without achieving this overarching goal control of the epidemic will not be achieved. Therefore due attention is needed to avert the current epidemics and diagnosis of cases.

**Electronic supplementary material:**

The online version of this article (10.1186/s12879-018-3214-6) contains supplementary material, which is available to authorized users.

## Background

Human immunodeficiency virus (HIV) infection remains the leading cause of morbidity and mortality throughout the world. Since the start of the epidemic, around 76.1 million people have become infected and 35.0 million people have died from AIDS (acquired immunodeficiency syndrome) related illnesses. Globally, in 2016 there were 36.7 million people living with HIV, 1.8 million new HIV infections, and 1 million AIDS related deaths [1]. Sub-Saharan Africa (SSA) contributed 76% of the total HIV-infected people, 76% of the total new HIV infections, and 75% of the total HIV/AIDS deaths in 2015 [2].

As one of the sub-Saharan country the case in Ethiopia is not different. It is characterized by a low-intensity, mixed epidemic and self-sustaining transmission with a prevalence of 1.1%. In 2016, there were 720,000 people living with HIV and 27,104 newly diagnosed cases. But only 67% of expected PLWH know their status and 59% of them were enrolled in highly active antiretroviral therapy (HAART) program, while significant proportion people living with HIV were died [1, 3].

To avert this problem; Ethiopia adopted the global 90–90-90 HIV prevention targets by 2020 which is part of strategies designed to eliminate HIV/AIDS epidemics by 2030. According to this target plan, by 2020, 90% of all people living with HIV will know their HIV status, 90% of all people with diagnosed HIV infection will receive sustained antiretroviral therapy and 90% of all people receiving antiretroviral therapy will have viral suppression. Therefore achievement of these targets by 2020 is helpful for elimination of AIDS epidemic in 2030 [4, 5]. However, achieving or approaching to achieve these targets highly depends on the trend of HIV infection in the previous years, the burden of the disease, commitment and capacity of the leaders and implementation of the designed strategies to achieve the target. Thus, HIV/AIDS indicators have high importance in indicating the extent of the problemthe effect of interventions and predicting the future values for monitoring and evaluating the progress of the target under the existing program interventions. In spite of these facts utilization of HIV/AIDS indicators for the aforementioned purposes is very low [6].

Moreover, HIV/AIDS indicator data were long been recorded and reported as raw data, no rigor analysis was done to assess the trend and current status of the problem as well as no prediction was done to evaluate the pattern of the achievements towards the 90–90-90 HIV/AIDS prevention goal at local or national level. Trend analysis has particular advantages to know the pattern we are heading to, predict the future and identify predictors of changes within a time frame.

Trend analysis to assess current HIV status, building model for predicting future HIV/AIDS indicator values and identifying predictors have greater importance. Thus, the evidence could help planners, implementers and aid organizations in the country and abroad to take evidence based actions. Therefore this analysis aimed to show trend of HIV/AIDS diagnosis, enrolment, treatment and outcomes along with most important other indicators.; Thereby health care system could take a lesson about how the pace of the program is going towards the achievement of the goal. Generally, the evidence will be used as baseline information for planning, monitoring and evaluating the program throughout the health care system.

## Methods

### Study design, settings and population

Time series analysis was conducted using international data bases of health metrics from 1990 to 2016 from UNAIDS, WHO and World Bank data bases to investigate trends of HIV/AIDS for the last 26 years in Ethiopia and its regions. Ethiopia is the second most populous country in Africa next to Nigeria, with a population estimated at 99,390, 000 in 2015 of which 83.86% live in rural areas. Ethiopia is a Federal state composed of 9 Regional states and two city Administrations [4, 7].

HIV/AIDS has been recognized since the mid-1980s in Ethiopia, the epidemic has remained a major public health problem, largely affecting people of productive and reproductive age. To overcome this problem, a wide HIV related programs tailored on provision of preventive care, support and treatment services were designed. As part of the responses, Federal HIV/AIDS Prevention and Control Office (FHAPCO) was established in 2002 to coordinate the programs. A regular surveillance has been carried out in recent years through surveys in antenatal clinics (ANC), Demographic and Health Surveys (DHS) and Behavioral Surveys (BSS) and future values were forecasted, which are some of the basic data sources of this research [4, 7].

### Study variables, sources of data and data collection procedure

United nation country-level data (data for each member state) is collected by specialized agencies of the United Nations (such as WHO for health data in general and UNAIDS for HIV/AIDS specific data), and other intergovernmental organizations such as World Bank and UNDP which compile data from many different sources and organize for decision making purpose.

The major sources of data for this research is particularly UNAIDS data base (http://aidsinfo.unaids.org/) which contains data related with HIV incidence, prevalence, test, treatment, prevention and outcomes in regional, sub-regional and national as well as with age and sex sub-classifications. Other agencies of united nation, WHO (http://www.who.int/healthinfo/statistics/en/), UNICEF, UNDP and World Bank (https://data.worldbank.org/) data bases were also used for abstracting variables not found in the aforementioned data base. In addition to these sources of raw data, published form of data were used from WHO and the Ethiopian ministry of health data bases.

Majority of the data sources were validated by the agency (UN), while some are direct reports submitted by member states from findings of national surveillance, national surveys, censuses, vital statistics, modeled prediction (forecasts) and large surveys/researches. Country-specific estimates were based on the available interagency estimates for which data is available since 1990 for many of the variables and back to 2010 for some other variables.

The study variables were selected purposively from UNAIDS Global AIDS Monitoring 2017 guide line [6]. The one which are proxy measures of the 90–90-90 goal, which have Public Health importance (widely used) and the one which have available data are selected. Finally, indicators like: Percentage and or number of People living with HIV who know their HIV status, People living with HIV on antiretroviral therapy, Retention on antiretroviral therapy at 12 months, percentage of People living with HIV who have suppressed viral load, AIDS mortality, Mother-to-child transmission of HIV, Preventing the mother-to-child transmission of HIV, HIV incidence and prevalence are used for assessing the trend of HIV and to predict (forecast) the achievement of the 90–90-90 HIV prevention target by 2020 goal.

Data were collected by investigators and their assistants who are experienced in data mining. Preformed document extraction and collection check list was used tp collect data. Data regarding initially selected study variables were collected from the identified data bases. Data entry was done using preformed data entry template designed on Microsoft excel. All indicators were defined according to their standard definition given by the source of the indicators (UNAIDS and WHO).

### Operational definition

According to the definitions of UNAIDS and World Health Organization the following definitions of terms are adopted [6].

- **People living with HIV who know their HIV status**: Percentage of people living with HIV who know their HIV status at the end of the reporting period
- **People living with HIV on antiretroviral therapy**: Percentage and number of adults and children on antiretroviral therapy among all adults and children living with HIV at the end of the reporting period
- **Retention on antiretroviral therapy at 12 months**: Percentage of adults and children living with HIV known to be on antiretroviral therapy 12 months after starting
- **People living with HIV who have suppressed viral loads**: Number and percentage of people living with HIV who have suppressed viral loads at the end of the reporting period
- **AIDS mortality**: Total number of people who have died from AIDS-related causes per 100,000 population
- **Mother-to-child transmission of HIV**: Estimated percentage of children newly infected with HIV from mother-to-child transmission among women living with HIV delivering in the past 12 months
- **Preventing the mother-to-child transmission of HIV**: Percentage of pregnant women living with HIV who received antiretroviral medicine to reduce the risk of mother-to-child transmission of HIV
- **HIV incidence**: Number of people newly infected with HIV in the reporting period per 1000 uninfected population

### Statistical analysis

After the data was obtained from different sources, it was compiled with excel, each variable was checked for completeness and consistency. Whenever a series of data was obtained from more than one source validation was done and either UNAIDS or WHO with the most recent one was used. Data was cleaned, coded and exported to STATA version 11 for Windows, and then exploratory data analysis was carried out.

The patterns of each selected indicators and their change were described numerically and graphically with line graphs plotted using points on the X-Y axis, where X is the time in year (mostly from 1990 to 2016) and Y is value of the selected indicator for each year in number or percent. Trend line equation that best fits the regression line was drawn using coefficient of determination. The rates of annual change of the indicators values were also estimated. Possible correlation between variables was assessed with a correlation matrix and ARIMA model. The best fit equations of the trend line of an indicator were used to predict the future values of the indicator for 2020 target and the predicted values were compared with the 90–90-90 by 2020 target values.

## Results

### HIV diagnosis, incidence and prevalence

The national HIV prevention and treatment programmes were effective in reducing incidence of HIV/AIDS infection at the national and regional levels through early diagnosis, treatment and care. However, still HIV is a public health problem in Ethiopia [3, 4].

**Fig. 1:**
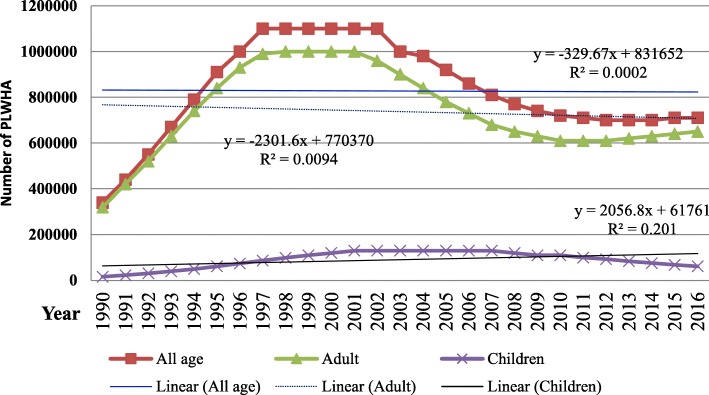
HIV prevalent cases for all age, adults and children of Ethiopia, 1990–2016

**Fig. 2:**
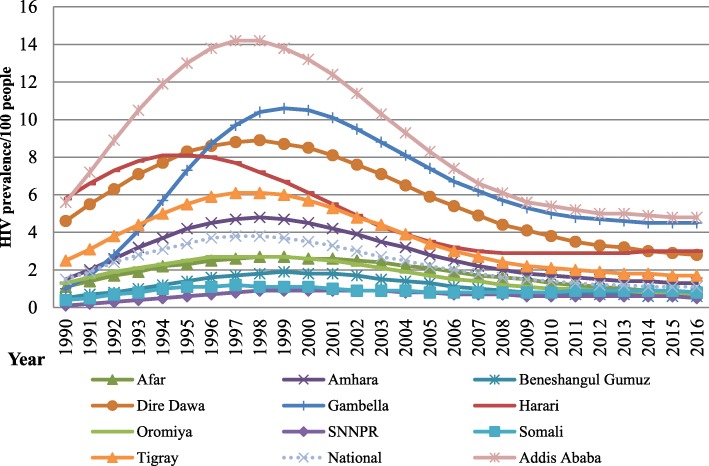
HIV prevalence among all age groups for national and regional value of Ethiopia, 1990–2016

In 2016, 67% (53–83%) of people living with HIV knew their status. In the same year there were an estimated 710,000 (570,000- 880,000) people living with HIV among the general population. Of which 650,000 (520,000-800,000) were adults and the remaining 62,000 (42,000-84,000) were children. The overall prevalence of HIV/AIDS is 1.1% among all ages. Since 2002, the annual number of HIV infected people in the country has declined by 35.5% from the peak level of 1.1 million (0.9 million-1.3 million). However, the rate of decline since 1990 was 330 cases per year among all ages and 2057 cases per year among children, which is too slow compared to the decline observed since 2002 (Figs. 1 & 2).

**Fig. 3:**
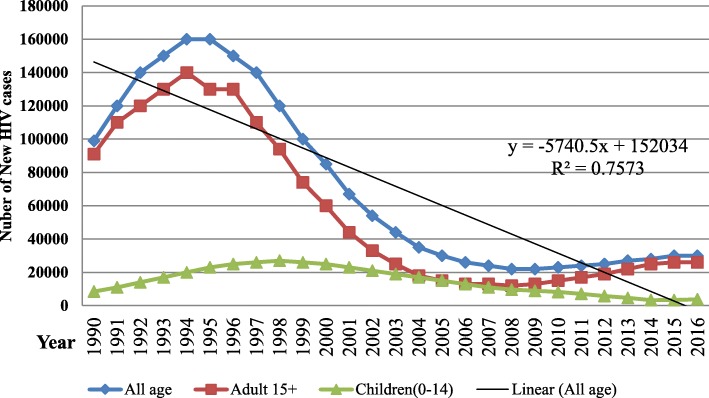
Number of HIV incident cases for all age, adults and children of Ethiopia, 1990–2016

**Fig. 4:**
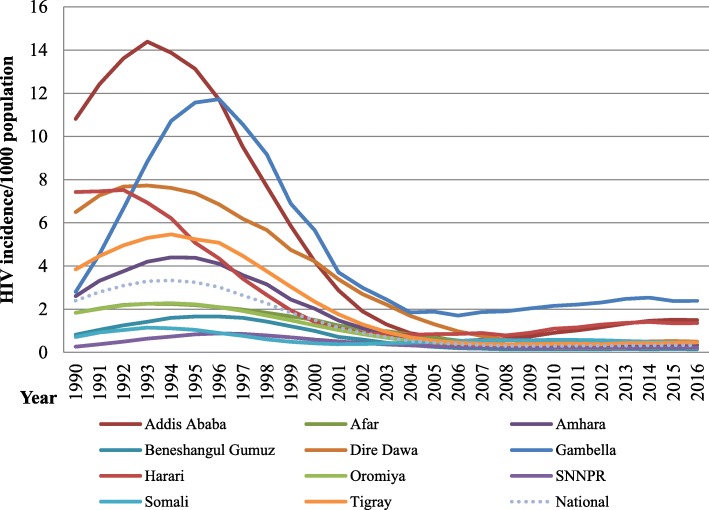
HIV incidence among all age groups for national and regional value of Ethiopia, 1990–2016

The pace of decline in new HIV infectionsis faster both among adults and children in all regions. In 1995 new HIV infection has reached a peak level. There were 160,000 new infections annually, onwards this point of time, it progressively decline and the new infection dropped by 81% to 30,000 (19,000-41,000) cases in 2016. The incidence of HIV infection has declined by 0.1352 per 1000 population since 1990 and reached 0.33 per 1000 population among all ages in 2016. Even though there is remarkable decline in incidence of HIV in all regions; the rate of decline and the current incidence revealed huge disparities among regions in Ethiopia (Figs. 3 & 4).

Regional trends in the annual number of new HIV infections and the prevalence density among all ages have varied. The incidence rate of 2.39/1000 population in Gambela Region, 1.5/1000 population in Addis Ababa city administration and 1.36/1000 population in Hareri region are by far higher than the national incidence. Similarly the prevalence of HIV in Addis Ababa (4.8%), Gambela (4.5%), Hareri (3%) and Dire dawa (2.8%) are by far Higher than the national prevalence and the global cut off point for generalized epidemic declaration (Fig. 4).

After continuous decline for decades, since 2008; HIV incidence began to rise by 10% from the level of 0.3/1000 population to 0.33/1000 population in 2016. At the same time the number of new infection increased by 36% from the level of 22,000 to 30,000 among all ages and doubled from 12,000 to 26,000 among adults. The rate of increment is higher in Gambela, Addis Ababa and Hareri regions. However, the number of new infection has reduced by 60% among children at national level (Figs. 3 & 4).

### HIV/AIDS treatment and care

**Fig. 5:**
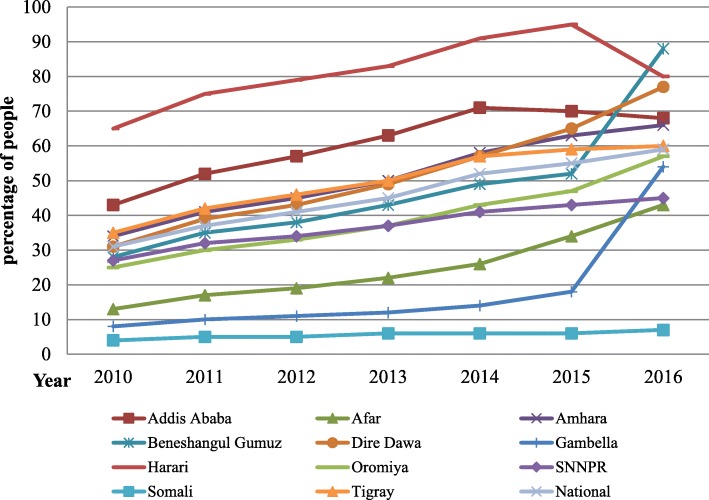
Percentage of people living with HIV receiving ARV for all age National and regional value of Ethiopia, 2010–16

The national scale-up of antiretroviral therapy has been highly accelerated to address patients who need HIV care. Nationally, 420,000 clients were on ART in 2016; which means 59% of all people living with HIV or 88% of people living with HIV who know their status were on treatment. Similarly 35% (23–47%) of children living with HIV and 61% (49–75%) of adults were receiving ART by 2016. The coverage was even higher in Benishangul, Harari, Dire Dawa, Addis Ababa and Amhara Regions. Since 2010, the coverage of antiretroviral treatment has increased by 4.6% per year. Within 6 years (2010–2016) the coverage of antiretroviral treatment coverage rises to 59% form 31% coverage since 2010. After implementation of test and treat program at the end of 2014, the coverage has raised significantly in most of the regions (Fig. 5).

In the same manner to the general population, the coverage of antiretroviral medicines provided to pregnant women living with HIV to prevent transmission to their children also rose from 25% (17–32%) to 69% (50–87%) over the same period. I.e. around 16,700 pregnant women were received ART among 24,000 women who need to have ART for PMTCT.

Higher treatment coverage along with better adherence (retention to 12 months on treatment) allowed patients to have suppressed viral load. In 2012, 90% of peoples receiving ART were retained for 12 months and 51% (41–63%) of all people living with HIV or 86% of people living with HIV on treatment has viral suppression in 2016. In addition, around 201,000 deaths (28,714 deaths each year) among the general population were averted with ART and 24,200 new infections were also averted through PMTCT since 2010.

### HIV /AIDS related mortality

**Fig. 6:**
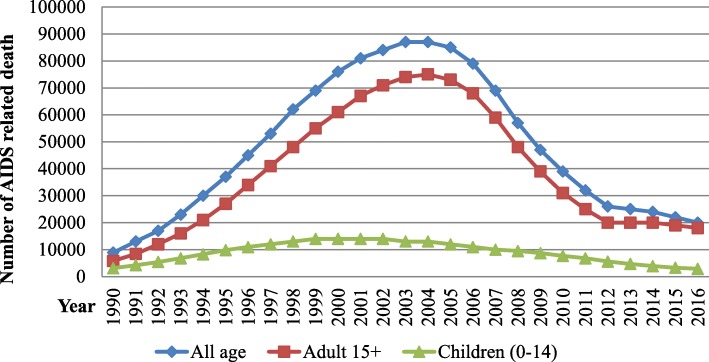
Number of AIDS related deaths among all ages, adults and children of Ethiopia, 1990–2016

Nationally, AIDS related death reached peak in 2003 with 87,000 (70,000-100,000) deaths. Since then, it gradually declined in subsequent years and reached 20,000 (13,000-31,000) deaths in 2016, which is 77% reduction from the maximum recorded AIDS related death in the country. The number of adults dying of AIDS-related illnesses has declined from 75,000 to 18,000 and the number of children (aged 0–14 years) dying of AIDS-related illnesses has been also declined by 79% in 14 years, from 14,000 in 2002 to 3900 in 2016. The trend of AIDS related death has a sharp rise from 1990 to 2002 and 2003 where it reached a peak among children and general population respectively, then stared to decline sharply to 2016s. However the decline of mortality among adults since 2012 was steady with only 1% decline (Fig. 6).

### Trends and prediction of the 2020 targets

**Fig. 7:**
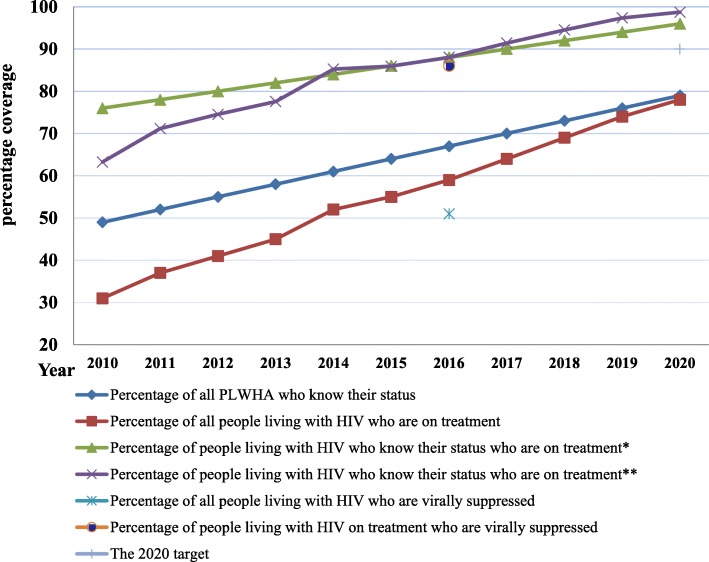
The 90–90-90 by 2020 HIV prevention targets and predicted values for Ethiopia

The values of the 2020 targets were forecasted based on the trend of indicators back to 2010 with the available data. Based on this prediction, 79% of people living with HIV/AIDS will know their status by the year 2020. Of which, about 96–99% will initiate ARV treatment by the same year. It is forecasted from the value of people who will be on treatment by 2020 (78.5%),proportion of people living with HIV who will know their status by 2020 (79%) and a direct forecast from the value that percentage of people living with HIV who know their status who are on treatment in 2015 (86%) and in 2016 (88%). But the percentage of people on ART who will have viral suppression is not forecasted due to the fact that it is nationally estimated only once in 2016, therefore comparison was made based on only this value (Fig. 7).

## Discussion

Within three decades, since first case of HIV infection was reported in Ethiopia, almost 1.93 million people have been infected with HIV and about 1.3 million (67.4% of infected peoples) have died of AIDS-related causes [8–10]. The national HIV prevention and treatment programmes has made considerable progress in addressing this unprecedented epidemic and averted many more new infection and AIDS related death. Since then, the burden of HIV/AIDS infection had declined at the national and regional levels through early diagnosis, treatment and care [4, 11–13]. However, still HIV is a public health problem in the country. In order to overcome this problem Ethiopia has adopted the 90–90-90 HIV prevention target by 2020 in 2014/5 in order to end AIDS epidemic by 2030 [4, 5].

In 1990s a substantial increment in the number of people living with HIV and new infections among all ages was observed throughout the country. These national HIV/AIDS metrics are in line with the global HIV epidemic pattern [9, 14]. Between the periods of 1990–1995 the number of new infection has sharply risen from 100,000–160,000 per year. It resulted in continuing large number of people living with HIV in 1997–2002, where nearly 1.1 million people were living with HIV in Ethiopia. Higher number of new HIV infection until the 1995 [9] and improved survival of people living with HIV/AIDS owing to the initiation of ART program [4, 15, 16] resulted in high prevalence of HIV until 2002. However, after the prevention programmes have expanded throughout the country, new infection and prevalence of HIV has declined remarkably.

The pace of decline in new HIV infection and prevalence is not even across different age groups and between regions. Among children, new infection has declined from 27,000 in 1998 to 3300 in 2014 and prevalent cases from 130,000 in 2007 to 62,000 in 2016. This is particularly resulted from wider use of improved ARV medicine regimens and improved PMTCT service [4, 16]. Moreover, The Global Plan towards the elimination of new HIV infections among children by 2015 and keeping their mothers alive which is implemented in Ethiopia as well, added strong achievement in the prevention program [17]. The decline of new HIV infection and prevalent cases however do not sustained after 2008 among adults, virtually the entire decline in HIV incidence has majorly resulted from reduction of infection among children [16, 17].

The trend of HIV prevalence depicts a heterogeneous epidemic in the country, with the highest prevalence regions being Addis Ababa (4.8%), Gambela (4.5%), Hareri (3%) and Dire dawa (2.8%), which is much higher than the national average prevalence and the level of generalized epidemic declaration. Similarly, the incidence of HIV in these regions is by far higher than the national average. The reason why HIV is more prevalent in these areas may be due to the fact that these are the major cities and trade routes of the country in which migrants from different corner of the country migrate for job and became highly vulnerable for HIV [4, 5, 13]. In addition to this the case of Gambela was explained with lower male circumcision rate in the region [18].

Besides remarkable reduction in HIV epidemics, the current epidemics observed since 2009 among adult is another challenge which needs a due attention. Since this period incidence of new HIV among all age in all regions rose sustainably as a result of increased new infection among adults which account for the vast majority of people newly infected annually. This trend is in line to the global trend of HIV which indicates the world is performing poorly in preventing sexually active people from acquiring HIV [5, 14]. Therefore, as new infection was prevented among children through prevention of Mother to child transmission, the global strategy should also address the sexual transmission of HIV among adults.

Nationally, coverage of ART treatment has expanded from 31% in 2010 to 59% in 2016 nearly doubled within 6 years. The estimated ART coverage in Ethiopia is comparable to the Eastern and southern Africa regional average of 60% and higher than the regional averages of Middle east and north Africa (24%), western and central Africa (35%), Asia and the pacific (47%), Latin America (56%) and Caribbean region (52%). While it is lower than the regional average of western and central Europe and North America of 78% coverage. This could be due to difference in access, retention and difference in implementation of the program [14, 19]. In Ethiopia, access is highly expanded in current few years, retention is higher (90%) and furthermore integration of the ART program with Health Extension package (HEWs) and involvement of Health Development Army (HDA) to HIV care services highly contributed. Yet, this increment in the coverage of HAART did not limit the new infection observed in recent years due to higher rate of HIV sexual transmission among adults [20].

Furthermore, implementation of test and treat program, strengthening of PMTCT program and achievement of higher retention in ART program resulted in suppression of viral load and averted many more new infections and AIDS related death [4, 14, 21]. The reduction of new infection among children that contributed for the reduction of HIV prevalence among the general population was basically a result of this PMTCT program. The ART program also has higher contribution in the prevention of transmission among adults [21]. The trend of coverage had dramatically increased in all regions since 2010, except for the last 1 year in Hareri and 2 years in Addis Ababa regions that could be related to the increase in prevalence of HIV in recent years.

The fact that HIV is one of the major causes of morbidity and mortality in Ethiopia was reported from previous studies [4, 5, 13, 22] as it was in many other developing countries [22]. Since 1990, 1.3 million people (50,000 people each year) were died from AIDS related cause. Such higher level of mortality was also seen in many of sub-Saharan countries where access to life saving HAART is low and the risk of HIV progression and related mortality is high [20, 21].

The trend of AIDS related death has a sharp rise from 1990 to 2002 and 2003 when it reached a peak among children and general population respectively and stared to decline sharply to 2016s level. Since then, the annual number of deaths from AIDS declined as well and a decade later it was almost a quarter when 18,000 people died in 2016. Much of the decline is due to steep reductions in new HIV infections among children with increased access to pediatric antiretroviral therapy and strengthening of PMTCT service [16, 20, 21]. The reduction of new HIV among adults appreciated since 1995 also contributed a lot for this achievement. Such a trend in reduction of AIDS related mortality was also achieved in the Caribbean, western and central Europe and North America, Asia and the Pacific and western and central Africa regions [20, 21].

Access to ART has direct impact on an individual’s risk of death, and on the country where one lives has a significant impact on death rates and life expectancy. Generally, early access to life-saving ART highly reduces AIDS-related death. Particularly in low-income countries and high HIV burden countries, annual burden of AIDS related death markedly increased due to low access of ART treatment. Besides early access, economic status of the clients also determines the mortality level of individuals related to HIV. In some lower income countries, people living with HIV have 10 to 20 times higher death rates than those living in some higher income countries [21].

In 2016, 67% of people living with HIV knew their status, 86% of people living with HIV who know their status were on treatment and 88% of people on treatment have viral suppression. This is by far greater than the regional average of 42, 83 and 73% for people who know their status, who are on treatment and who have viral suppression respectively in Western and Central Africa WHO region [21]. Based on the trend prediction, by 2020, 79% of people living with HIV will know their status and 96–99% of people knowing to have HIV infection will initiate a treatment by the same year.

Ethiopia has made Remarkable progress towards achieving the 90–90–90 targets evidenced that attainment of the 90–90-90 target by 2020 is both feasible and reachable if gaps across the HIV testing and treatment cascade are promptly addressed. However, the first 90% target (90% of people living with HIV knowing their status) is not achievable in Ethiopia by 2020. While the second 90% target, (to treat 90% of people who know their status) will be achieved 3 years before the specified date. The third target (90% of viral suppression among those who are on treatment) was not predicted for its 2020 value because it is estimated only once in 2016 nationwide. However even by this point, around 86% of people on ART have viral suppression near to achieve the 90% target by 2016 4 years before the actual time frame.

The findings of this study might suffer from the fact that it is retrospective study and based on records; the reliability of the recorded data couldn’t be ascertained and potential bias associated with estimation is there. Moreover, the forecasted values from the trend may change through time due to change in intervention programs. The determinants of each outcome and the trend was not addressed which may have influence as well.

## Conclusion and recommendation

After remarkable decline in incidence and prevalence of HIV infection among all ages, it started to increase in the last few years. The incidence of new infection and prevalent cases among adults since 2009 started to increase remarkably due to suppressed prevention programs, while sustained decline in incidence among children was achieved through PMTCT program and it remarkably contributed to the national level decline of the epidemics. Increased coverage of ART in the last decade has remarkably declined AIDS related death and averted many more new infections. Ethiopia has made Remarkable progress towards achieving the 90–90–90 targets in which the second and third 90% targets for treatment and viral suppression are achievable, while the first target for HIV diagnosis is not achievable and without achieving this overarching goal control of the epidemic will not be achieved. Therefore due attention is needed to avert current increasing incidence of HIV among adults and it is also important to diagnoses those who have the virus which is base for the other targets.

## Additional file

Additional file 1: Trend of HIV/AIDS for the last 26 Years and Predicting Achievement of the 90-90-90 HIV Prevention Targets by 2020 in Ethiopia: A Time Series Analysis. (XLSX 18 kb)

## Abbreviations

AIDS: Acquired Immune Deficiency Syndrome
ART: Antiretroviral therapy
DHS: Demographic and Health Surveys
FHAPCO: Federal HIV/AIDS Prevention and Control Office
HAART: highly active antiretroviral therapy
HIV: Human immune deficiency virus
PMTCT: Prevention of mother to child transmission of HIV
UNAIDS: United Nations Programme on HIV/AIDS
UNDP: United Nations Development Program
UNICEF: United Nations Children’s Fund
WHO: World Health Organization
